# Transcriptional Profiles Uncover *Aspergillus flavus-*Induced Resistance in Maize Kernels

**DOI:** 10.3390/toxins3070766

**Published:** 2011-06-29

**Authors:** Meng Luo, Robert L. Brown, Zhi-Yuan Chen, Abebe Menkir, Jiujiang Yu, Deepak Bhatnagar

**Affiliations:** 1 Department of Plant Pathology and Crop Physiology, Louisiana State University Agricultural Center, Baton Rouge, LA 70803, USA; Email: meng.luo@ars.usda.gov (M.L.); zchen@agcenter.lsu.edu (Z.-Y.C.); 2 Southern Regional Research Center, United States Department of Agriculture-Agricultural Research Service, New Orleans, LA 70124, USA; Email: jiujiang.yu@ars.usda.gov (J.Y.); deepak.bhatnagar@ars.usda.gov (D.B.); 3 International Institute of Tropical Agriculture, Oyo Road, PMB 5320, Ibadan, Nigeria; Email: a.menkir@cgiar.org

**Keywords:** *Zea mays*, *Aspergillus flavus*, imbibed kernels, aflatoxin, resistance genes

## Abstract

Aflatoxin contamination caused by the opportunistic pathogen *A. flavus* is a major concern in maize production prior to harvest and through storage. Previous studies have highlighted the constitutive production of proteins involved in maize kernel resistance against *A. flavus*’ infection. However, little is known about induced resistance nor about defense gene expression and regulation in kernels. In this study, maize oligonucleotide arrays and a pair of closely-related maize lines varying in aflatoxin accumulation were used to reveal the gene expression network in imbibed mature kernels in response to *A. flavus*’ challenge. Inoculated kernels were incubated 72 h via the laboratory-based Kernel Screening Assay (KSA), which highlights kernel responses to fungal challenge. Gene expression profiling detected 6955 genes in resistant and 6565 genes in susceptible controls; 214 genes induced in resistant and 2159 genes induced in susceptible inoculated kernels. Defense related and regulation related genes were identified in both treatments. Comparisons between the resistant and susceptible lines indicate differences in the gene expression network which may enhance our understanding of the maize-*A. flavus* interaction.

## 1. Introduction

*Aspergillus flavus* is not only a saprophytic fungus, but an opportunistic pathogen which invades susceptible hosts such as maize, cottonseed, tree nuts, and peanuts [1]. Aflatoxin contamination caused by *A. flavus* is a major concern in maize production prior to harvest and through storage [2,3,4]. Aflatoxins are secondary metabolites of this fungus which can be highly toxigenic and carcinogenic to humans or animals consuming contaminated food or feeds [5,6,7]. Significant research has been devoted to developing ways of controlling aflatoxin contamination of crops. Understanding the molecular mechanisms involved in the interaction between *A. flavus* and maize kernels would aid the development of strategies to interrupt the aflatoxin contamination process. 

The morphological process and the molecular mechanisms of *A. flavus* involved in maize kernel invasion have been widely observed and discussed [8]. Numerous fungal genes have been shown to be involved in the invasion process and in aflatoxins biosynthesis [9,10]. However, identifying genetic resistance mechanisms in maize kernels of aflatoxin-resistant lines, and under varied environmental conditions, can be very challenging. To control the environmental effects, better ascertain kernel genetic differences between genotypes and assist field screening in maize breeding, the laboratory-based Kernel Screening Assay (KSA) was developed [11,12]. The KSA uses mature kernels inoculated with *A. flavus* to quantify aflatoxin accumulation, therefore, highlighting the phase of kernel development in the field where aflatoxin increases. This technique speeds-up aflatoxin assessment and eliminates escapes. The KSA correlates well with field trial results [12,13,14], and is a primary technique used to screen germplasm in a collaborative project for breeding aflatoxin-resistant maize lines between the International Institute of Tropical Agriculture (IITA) and the Southern Regional Research Center (SRRC) of the USDA-ARS [15]. Six aflatoxin-resistant inbred lines were released to the public through this collaboration [14]. 

Over the past twenty years, a number of resistant maize lines with low aflatoxin accumulation levels have been identified or developed [14,15,16,17,18]. While maize hybrids with improved resistance to *A. flavus* infection and aflatoxin biosynthesis may be in commercial use, the levels of resistance are not yet adequate to prevent unacceptable aflatoxin concentrations (FDA has limits of 20 ppb, total aflatoxins on interstate commerce of food and feed, and 0.5 ppb of aflatoxin M_1_ on the sale of milk) [17]. To make use of maize germplasm with greater resistance that are available now or in the future, efficient biomarkers are needed [17].

Plants have defenses against most phytopathogens through recognition and the triggering of a wide range of defense responses, including the reprogramming of cellular metabolism, the accumulation of barrier-forming substances, and the production of antimicrobial compounds, which act directly to prevent pathogen invasion [19,20]. Despite impressive advances in knowledge concerning defense mechanisms in vegetative plants [19,21,22], little is known about molecular mechanisms of plant seeds for defending against fungal infection. This is especially the case regarding infection by facultative pathogen, *A. flavus.*

Previous studies indicate that both constitutive and induced resistance are involved in maize kernel defense against *A. flavus* infection [16,23]. Comparative proteomics has identified numerous constitutive resistance-associated proteins (RAPs) in mature kernels [24,25], presuming their resistant function in aflatoxin contamination. Meng *et al.* [26] analyzed the gene expression profile of aflatoxin-resistant inbred Tex6 during kernel development using microarray analysis and found that RAP genes were significantly expressed at the late developmental stage. In that study, kernels in developing ears were used to observe induced resistance in response to *A. flavus* infection introduced by a non-wound inoculation method. However, consistent gene profiles were unable to be acquired due to variation between experimental replicates caused by factors such as the kernel developmental stage, the environment, and/or *A. flavus* inoculation methods. The purpose of the present study is to determine gene expression differences between aflatoxin-resistant and -susceptible maize lines in response to *A. flavus*’ challenge. This may highlight the presence of inducible resistance factors to complement constitutive factors previously identified through comparative proteomics. Employing gene expression analysis can also overcome the limitations of protein analysis such as the expensive costs involved in identifying a complete proteome and the lack of visibility of some lowly-expressed protein spots, which potentially limits the detection of important proteins. To minimize the effect of different genetic backgrounds on gene expression, two closely-related inbred lines, Eyl25 and Eyl31, were used; these were derived from a cross between two resistant lines, 1368 and GT-MAS:gk, in the SRRC-IITA collaborative project [14,15]. Of the two lines, Eyl25 is aflatoxin-resistant (R), and Eyl31 is susceptible (S). To eliminate the effects caused by using developing kernels in field trials, imbibed mature kernels (under KSA conditions) were used in this research. The KSA protocol involves inoculating kernels with *A. flavus* and incubating them at 31 °C and 100% humidity. This method attempts to create an “ideal” environment for maize kernel infection and subsequent aflatoxin production. To acquire gene expression profiles, oligonucleotide microarrays developed by the Maize Oligonucleotide Array Project [27] were used.

## 2. Materials and Methods

### 2.1. Plant Treatment

Dry mature maize kernels of aflatoxin-resistant Eyl25 and of -susceptible Eyl31 used in this study were provided by Dr. Abebe Menkir of IITA of Ibadan, Nigeria. The *A. flavus* strain used was the same as in all other studies performed in this lab, AF13 (ATCC 96044; SRRC 1273). Kernels were sterilized and inoculated with *A. flavus* as described in the KSA protocol [12]. Noninoculated kernels served as controls. For each treatment, 40 kernels were used. After 72-h incubation at 31 °C and 100% humidity, kernels in each treatment were bulked and washed three times using 0.02% Triton X, each time for 3 min, followed by rinsing with DD H_2_O to remove *A. flavus* growth from kernel surfaces. Kernels were then dried using absorbing paper, and frozen using liquid nitrogen. All kernels were kept at −70 °C until RNA extraction. A parallel experiment to assess fungal colonization levels and aflatoxin accumulation in inoculated kernels was conducted according to the KSA protocol. After 72 h incubation with *A. flavus*, colonization of kernels was classified on a 5 level system based on the percentage of kernel surface colonized: 1 = 1–20% of the surface colonized; 2 = 21–40%; 3 = 41–60%; 4 = 61–80%; 5 = 81–100%. After 7 days incubation, aflatoxin levels in inoculated kernels were quantified using a FluoroQuant Aflatest kit (Romer, Union, MO). 

### 2.2. RNA Isolation and Probe Labeling

Total RNA was extracted from seed using TRIzol reagent (Invitrogen, Carlsbad, CA). All RNA samples were treated with DNase (Qiagen, Valencia, CA) to remove DNA, and purified with the RNeasy system (Qiagen). RNA quantity and quality were assessed with a Nanodrop spectrophotometer (Nanodrop Technologies, Montchanin, DE). 

Fluorescent dye Cy3 and Cy5 labeled probes were prepared using the indirect labeling method of cRNA according to the protocol provided by The Maize Oligoarray Project [28]. A total of 6 μg of aminoallyl-cRNA were needed for each probe labeling. The aminoallyl-cRNA was synthesized and amplified using the RNA amplification system (Ambion, Austin, TX). Mono-reactive dyes Cy3 and Cy5 (Amersham, Piscataway, NJ, USA) were coupled to aminoallyl-cRNA from differently treated samples. The un-incorporated free dyes were removed with the RNeasy MinElute cleanup kit (Qiagen). 

### 2.3. Microarray Experimental Design and Data Analysis

Maize 46k 70-mer oligonucleotide arrays (Maize Oligonucleotide Array Project, version 1.0 [27]; were used in this study. Hybridization of slides was performed according to manufacturer’s instructions [28]. Kernels of inbred Eyl25 and Eyl31were bulked respectively from several individual ears before being shipped to the U.S. Therefore, no biological replicates were designed in the microarray experiment, only technical replicates based on pooling samples. A study on the utility of pooling biological samples in microarray experiments demonstrated that this method would not adversely affect most differentially expressed genes [29]. Our study was of a population phenotype (resistant or susceptible) and not of individuals within those populations and, therefore, appropriate for the pooling method. A direct comparison design was applied, which included Eyl25 Inoculated/Control, Eyl31 Inoculated/Control, Eyl25 Control/Eyl31 Control, and Eyl25 Inoculated/Eyl31 Inoculated. In each comparison, 4 technical replicates were used, including two dye swaps. Hybridized slides were scanned using a Genepix 4000B Scanner (Molecular Devices, Sunnyvale, CA, USA), and hybridization images were analyzed using GENEPIX 6.0 software. Signal values were initially normalized during the image scanning process by adjusting the photomultiplier tube (PMT) based on the average ratio between two channels. To eliminate the cross hybridization effect of *A. flavus* genes in the maize microarray hybridization under high PMT, the saturated spots ratio was set at 0.005%. This insured that the intensity of *A. flavus* genes would not affect maize gene expression results. 

Microarray data were analyzed using GeneSpring GX 10.0 software (Silicon Genetics, Redwood City, CA, USA). Two criteria were used for selecting positive spots, mean (Signal-Background) >400 unit as expression intensity filter, and the occurrence of at least two spots in the four replicates. These filters were imposed to remove genes with very minor differential expression or genes with little evidence for expression. Data normalization was performed using a LOWESS (locally weighted regression) algorithm. To identify statistically significant genes, a one-way ANOVA on the normalized data was performed using a *T*-test and *p*-values lower than 0.05 as criteria. Furthermore, a fold change analysis of significance was performed to address the magnitude of change of statistically significant genes.

### 2.4. Microarray Data Validation by qRT-PCR

Twenty-four genes with expression patterns of up-regulation, down-regulation, or no-change in the microarray analysis were selected for quantitative analysis using one-step qRT-PCR. Total RNA from above samples were treated with DNase (Qiagen), and subsequently purified with an RNeasy Cleanup Kit (Qiagen). Three technical replications were performed for each sample to assess the reproducibility, and the mean of the three replicates was used to calculate relative expression quantitation. One-step qRT-PCR was performed using the QuantiFast SYBR green RT-PCR kit (Qiagen) according to the manufacturer’s instructions. The total volume of the reaction was 20 μL which consisted of SYBR green RT-PCR master mix, QuantiFast RT mix, and 1 μM of each primer. Gene-specific primers were designed using Primer Express 3.0 (Applied Biosystems, Foster City, CA, USA) and amplicons were between 100–150 bp. The PCR assay was carried out using the Stepone Real-Time PCR System (Applied Biosystems). Cycling parameters were set according to the recommendation of QuantiFast SYBR green RT-PCR kit as: Reverse transcription at 50 °C for 10 min; PCR initial activation of DNA polymerase at 94 °C for 5 min, followed by 40 cycles at 95 °C for 10 s, and 60 °C for 30 s. At the end of the PCR final cycle, melting curves were run immediately to determine if measurements were influenced by primer-dimer pairs. 

The amplification curve was generated after analyzing raw data, and the cycle threshold (CT) value was calculated based on the fluorescence threshold of 0.01. The expression of the alpha-5 tubulin gene in kernels was very constant as demonstrated by micraorray and real-time PCR analysis, and was used as an internal reference in this study; primers were 5'-CTTGACATCGAAAGGCCAAC as the forward primer and 5'-CAAGGTTGGTCTGGAACTCAG as the reverse primer. A Student’s test and the “delta-delta CT” (2^−ΔΔCT^) mathematical model [30] were used for description and comparison of the relative quantification of gene expression between samples. Fold change of a target gene in the test sample was represented by *R* = 2^−ΔΔCT^, where ΔΔCT = ΔCT test sample-ΔCT reference sample, ΔCT sample = C(T)test gene-C(T)reference gene. The comparative result of a target gene in test sample and reference sample was described by statistical significance (*P* < 0.05) and fold change. 

## 3. Results

### 3.1. Aflatoxin Accumulation in Inoculated Kernels

Aflatoxin levels in inoculated kernels of aflatoxin-resistant maize line Eyl25 (R) and of-susceptible line Eyl31 (S) are shown in Table 1. Eyl25 demonstrated the same level of aflatoxin accumulation as the resistant check, while Eyl31 accumulated levels that exceeded both Eyl25 and the susceptible check.

**Table 1:** Pedigrees of Eyl25(R) and Eyl31(S) and aflatoxin accumulation in kernels.

Genotype	Pedigree	Aflatoxin* (ppb)
Eyl25	(1368xGT-MAS:GK)-8-1-1-4-B-B-B-B-B	315.1 c
Eyl31	(1368xGT-MAS:GK)-8-1-1-3-B-B-B-B-B	14112.5 a
MI82 (Resistance reference)	-	209 c
P3142 (Susceptible reference)	-	3298 b

* Mean values followed by the same letter are not significantly different by LSD test.

### 3.2. Fungal Colonization of Inoculated Kernels

After 72 h incubation of *A. flavus* inoculated kernels under KSA conditions, *A. flavus* colonization on kernel surfaces was observed, and the colonization level, characterized. Results indicated that all S kernels were colonized by *A. flavus*, and that the colonization levels of 53.3% of the inoculated kernels were rated >3 (60% surface colonized). However, 36.6% of the R inoculated kernels had visible fungal colonization, but none were rated >2 (40% surface colonized).

### 3.3. Defense-Related Genes in R and S Controls

To understand the gene expression profile in noninoculated kernels under KSA conditions, controls of the R and S genotypes were compared. According to the selective criteria of gene expression based on spot signal intensity on the microarray, 6955 genes (non-redundant IDs) in R and 6565 in S were detected. Of the total 8075 non-redundant expressed genes in both R and S controls, 5454 are contained within the expression overlap in the two controls, which is about 80% of the expressed genes in each genotype. Although the genetic similarity between Eyl25(R) and Eyl31(S) is 87.5% (Table 1), there are approximately 20% of the genes expressed differentially at the transcript level in each line. 

An important concern of this study was defense-related genes. Therefore, based on a gene ontology (GO) search in the maize biological process [31], defense-related genes were identified, and classified into six categories (Table S1). Results indicated that many pathogenesis-related (PR) genes were expressed in both R and S and that genes from most of the 17 PR families were observed [32]. Among the PR families, different members were detected in several families, such as in beta-1,3-glucanase, chitinase, lipid-transfer protein, and peroxidase families. However, expression values for members could be significantly different. For example, for nonspecific lipid-transfer protein in R, the value for member MZ00041610 was 41131.0, however, for member MZ00041203 the value was 512.5. Some biotic stress-related genes, related to pathogen recognition and signal transduction were detected in both genotypes. These include Avr9/Cf-9 rapidly elicited protein, mlo2 protein and receptor-like kinase Xa21-binding protein 3. Of all the defense gene categories, abiotic stress-related genes contained the most components. The stresses involved include heat, cold, salt, drought, wound, and UVB. Of the stress-related genes, heat shock protein (HSP) and glycine-rich protein were the families with the most members. From the survey of hormone-related genes, abscisic acid, auxin and ethylene contained the most genes involved in hormone synthesis and in response to hormones in both genotypes, but gibberellin had the fewest genes. The jasmonate induced gene (MZ00014430) and cytokinin inducible protein were only found in R. With regard to antioxidant and secondary metabolism genes, both genotypes expressed similar components. 

### 3.4. Differentially Expressed Genes in the Comparison between R and S Controls

**Figure 1:**
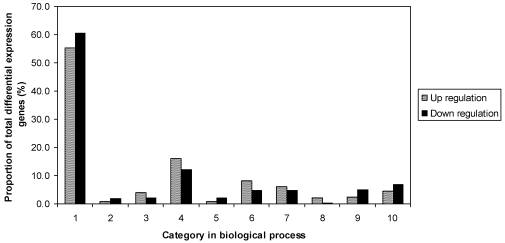
Functional categories of differentially expressed genes in the comparison of noninoculated Eyl25(R) with noninoculated Eyl31(S). 1 biological process unknown; 2 catabolism; 3 cell fate and development; 4 metabolism; 5 protein bio-synthesis; 6 protein fate; 7 response to stress; 8 signal transduction; 9 transcription; 10 transport.

To identify differences between R and S controls, gene expression profiles of the two were compared. The criteria for significant difference were set as *P* value <0.05, and fold change >2. Of the total 8075 non-redundant expressed genes in the R and S controls, there were 530 genes that were significantly different between R and S, including 248 up-regulated, and 282 down-regulated. The fold changes of differentially expressed genes were between 2 and 45.9. Results indicated that genes were distributed in all listed functional categories of biological processes (Figure 1). The largest proportion of genes were in the unknown category, including 55.2% of up-regulated and 60.6% of down-regulated genes. R had more genes involved in metabolism, protein fate, response to stress, and signal transduction. However, S had more genes involved in transcription and transport.

Defense-related genes with a significant difference between the R and S controls were also revealed. Of the six listed categories (Table S2), abiotic stress-related genes and pathogenesis-related genes comprised the majority, but their proportions and components were different in the two genotypes. There were more abiotic stress-related genes up-regulated in S than in R, but more than 50% of the genes belonged to HSP family. The HSP34 (MZ00035042) and HSP17.2 (MZ00031854) were the top two in fold-change ranking, which were 32.9 and 21.9 folds higher respectively in S than R. However, there were more PR genes in R than in S, with several members belonging to the chitinase family; no chitinase member was down-regulated in R. The PR gene with the greatest significant difference between the two genotypes was PR-4 (MZ00043659), which was 36.3 fold higher in R than in S. Of the antioxidant genes, all 5 up-regulated genes in S belonged to the glutathione S-transferase (GST) family. However, in R, catalase 3 (MZ00042638) was also up-regulated along with GST family member, GST 41 (MZ00026611). Based on GO search, several annotation unknown genes were classified as disease resistant, and were up-regulated either in R or in S. An example is gene MZ00019113, which was up-regulated by 14.5 fold in R. For the hormone related genes and secondary metabolism genes, fewer differentially expressed genes were observed in either genotype. 

### 3.5. Gene Expression in Kernels in Response to A. flavus’ Challenge

**Figure 2:**
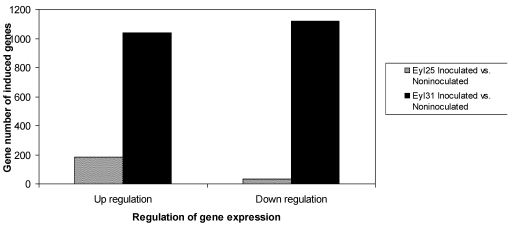
Survey of differentially expressed genes in *A. flavus* inoculated Eyl25(R) and Eyl31(S) kernels after 72 h incubation.

**Figure 3:**
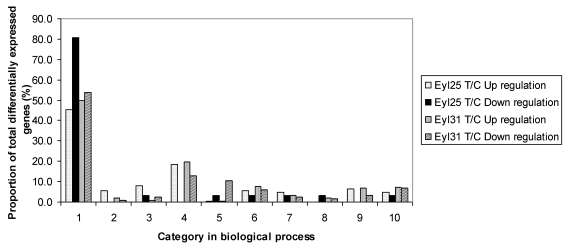
Proportion of differentially expressed genes among functional categories in the comparison among *A. flavus* challenged Eyl25(R), Eyl31(S), and noninoculated controls. 1 biological process unknown; 2 catabolism; 3 cell fate and development; 4 metabolism; 5 protein biosynthesis; 6 protein fate; 7 response to stress; 8 signal trans-duction; 9 transcription; 10 transport. T = inoculated; C = noninoculated.

The differentially expressed transcriptional profiles in the two genotypes were analyzed separately using microarrays. Results indicated that 214 genes in R and 2159 genes in S were induced compared to controls (Figure 2). Although R had fewer differentially expressed genes, it contained a higher proportion of up-regulated genes. Based on GO search of biological processes, the comparison of differentially expressed genes between R and S was conducted (Figure 3). Results showed that they were distributed in all listed categories; biological process unknown had the majority, followed by metabolism. 

**Figure 4:**
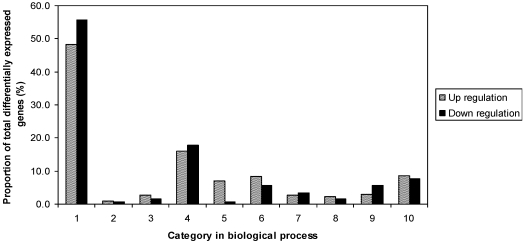
Proportion of differentially expressed genes among functional categories in the comparison of inoculated Eyl25(R) with inoculated Eyl31(S). 1 biological process unknown; 2 catabolism; 3 cell fate and development; 4 metabolism; 5 protein biosynthesis; 6 protein fate; 7  response to stress; 8 signal transduction; 9 transcription; 10 transport.

To study the difference between resistant and susceptible genotypes in response to fungal challenge, the two inoculated samples of R and S were compared in an independent experiment. A total of 1376 differentially expressed genes were observed, including 689 up-regulated and 687 down-regulated. The fold-changes of gene expression were between 2 and 125.8. Results also indicated that genes were distributed in all listed functional categories of biology processes (Figure 4). R had more genes in protein biosynthesis, protein fate, catabolism, cell fate and development, signal transduction, and transport. However, S had more in metabolism, stress related and transcription categories.

### 3.6. Defense Genes in Inoculated R and S

Defense-related genes were significantly expressed (*P* < 0.05, 2 fold change) in both inoculated resistant and susceptible genotypes, especially in S (Table S3). Of the up-regulated genes, PR and abiotic stress-related genes comprised the majority in both R and S, and the induced PR genes included most of the 17 PR families. However, the gene members and their expression levels in each family could be different. Chitinase, for example, had 12 members in R, and 14 in S. The maximum fold-change of the chitinase gene was 6 (MZ00043658) in R, but 18.3 (MZ00043035) in S. The maximum fold-change for defense genes in R was 6 for chitinase (MZ00043658), and only 3 defense genes were more than 5 fold different. The maximum fold-change in S was 85.7 for polyphenol oxidase (MZ00015021), and 10 defense genes were more than 10 fold different. The investigation also indicated that no PR gene was down regulated in inoculated R, but several were in S (Table S3).

**Figure 5:**
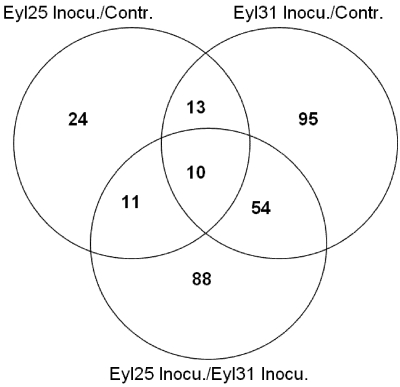
Venn diagram analysis for defense related genes for *A. flavus*-inoculated experiments involving Eyl25(R) and Eyl31(S).

Differences in defense-related genes were also compared between the inoculated samples of R and S (Table S4). Of the significantly expressed genes, several up-regulated genes, which were up-regulated in the comparison between noninoculated R and S, were also up-regulated in the R and S inoculated comparison. Examples of these were dehydration-responsive protein RD22 precursor (MZ00057294), glycine-rich protein (MZ00016231), pathogenesis-related protein 4 (MZ00043659), auxin-regulated like protein (MZ00042957), gibberellin-stimulated transcript 1 like protein (MZ00014890), 1,3-beta-glucanase (MZ00030174), nonspecific lipid-transfer protein precursor (MZ00041611), and Zeamatin precursor (MZ00017927). These results suggest that in the comparison between the two inoculated samples, differentially expressed genes were caused by both fungal challenge and having a different genetic background. To identify the differentially expressed genes caused by *A. flavus*’ challenge, and eliminate those caused by genetic background, Venn diagram analysis based on gene ID comparison was conducted between the inoculated R, the inoculated S, and the inoculated R/inoculated S (Figure 5). Results indicated that 75 defense-related genes were in response to *A. flavus*, and the remaining 88 genes were different due to genotype. Results also indicated that 23 defense genes were expressed in both inoculated resistant and susceptible genotypes. Of 75 defense-related genes (Table 2), more were up-regulated in S, especially PR genes.

**Table 2:** Significantly different induced genes in the comparison between inoculated Eyl25(R) and inoculated Eyl31(S) *. The direction of regulation comparisons is in R relative to S.

Gene ID	Fold Change	Regulation	Putative_Annotation
**Abiotic Stress Related Gene**			
Z00015715	2.82	up	OSJNBa0027O01.6
MZ00016855	6.38	up	salt-inducible protein kinase
MZ00017506	2.06	up	heat shock factor RHSF13-like
MZ00019961	3.18	up	unknown protein
MZ00025219	2.60	up	unnamed protein
MZ00026333	3.92	up	Emb5 gene
MZ00026695	3.75	up	nin one binding protein
MZ00027101	3.02	up	pseudouridylate synthase-like
MZ00027827	6.76	up	At4g08790/T32A17_100
MZ00028039	2.45	up	Late embryogenesis abundant protein EMB564
MZ00028141	2.46	up	Hsp70 binding protein
MZ00046743	19.14	up	wound inductive gene
**Antioxidant Gene**			
MZ00042868	3.02	up	glutathione transferase
MZ00015127	3.33	up	Hydroxyacylglutathione hydrolase cytoplasmic (Glx II)
MZ00014089	2.10	up	superoxide dismutase (Cu-Zn) 2
MZ00014859	2.43	up	glutathione S-transferase GST 18
**Biotic Stress Related Gene**			
MZ00018774	9.59	up	leucine-rich repeat-like protein
**Hormone Related Gene**			
MZ00043144	2.91	up	ABI3-interacting protein 2
**Pathogenesis Related Gene**			
MZ00017927	2.16	up	Zeamatin precursor
MZ00024296	2.20	up	2-oxoglutarate-dependent oxygenase
MZ00025038	2.51	up	pathogenesis-related protein 4
MZ00041277	2.95	up	chitinase
MZ00041611	4.71	up	Nonspecific lipid-transfer protein precursor (LTP)
MZ00042393	3.84	up	oxidase
MZ00043179	2.43	up	subtilisin/chymotrypsin inhibitor
MZ00043658	2.32	up	pathogenesis-related protein 4
MZ00043659	2.31	up	pathogenesis-related protein 4
MZ00043978	2.13	up	thionin like protein
**Secondary Metabolism**			
MZ00014812	2.47	up	cinnamyl alcohol dehydrogenase
**Abiotic Stress Related Gene**			
MZ00004118	2.42	down	multiple stress-responsive zinc-finger protein
MZ00015403	6.34	down	alcohol dehydrogenase ADH
MZ00016824	3.18	down	unknown protein
MZ00015918	4.37	down	unknown protein
MZ00026561	2.23	down	NA
MZ00036743	2.90	down	adhesive/proline-rich protein
MZ00037469	2.12	down	probable lipase
MZ00041634	5.14	down	adhesive/proline-rich protein
MZ00044463	2.48	down	multiple stress-associated zinc-finger protein
MZ00042137	3.80	down	phosphate-induced protein 1-like protein
**Antioxidant Gene**			
MZ00041713	15.90	down	glutathione S-transferase GST 8
**Biotic Stress Related Gene**			
MZ00028198	2.82	down	receptor-like kinase Xa21-binding protein 3
MZ00029329	13.99	down	receptor-like kinase
MZ00036884	3.53	down	Probable disease resistance protein At5g04720
MZ00043958	2.52	down	receptor-like protein kinase 1
**Hormone Related Gene**			
MZ00018872	10.09	down	acc synthase
MZ00027365	3.89	down	ethylene-forming enzyme
MZ00014879	2.03	down	auxin response factor 2
MZ00030445	2.42	down	ethylene-responsive factor-like protein 1
MZ00030984	3.30	down	chitin-inducible gibberellin-responsive protein
**Pathogenesis Related Gene**			
MZ00000977	5.45	down	antifungal thaumatin-like protein
MZ00004170	3.89	down	chitinase III
MZ00013547	2.65	down	thaumatin-like protein
MZ00015469	11.38	down	peroxidase
MZ00015553	3.82	down	Glucan endo-1,3-beta-glucosidase precursor
MZ00019543	3.30	down	peroxidase
MZ00020250	2.30	down	peroxidase
MZ00026196	8.92	down	peroxidase
MZ00026392	7.12	down	Bax inhibitor-1 (BI-1)
MZ00031167	2.66	down	antifungal zeamatin-like protein
MZ00035052	2.26	down	pathogenesis related protein-5
MZ00036117	2.38	down	thaumatin-like protein
MZ00037253	5.39	down	subtilisin/chymotrypsin inhibitor
MZ00041005	7.89	down	subtilisin/chymotrypsin inhibitor
MZ00041326	3.01	down	Bowman-Birk serine protease inhibitor
MZ00041327	6.10	down	Bowman-Birk type trypsin inhibitor (WTI)
MZ00041768	2.25	down	polyphenol oxidase
MZ00043035	5.66	down	chitinase PRm 3
MZ00043996	14.27	down	Bax inhibitor-1 (BI-1)
MZ00044200	2.82	down	beta-1,3-glucanase
**Secondary Metabolism Related Gene**			
MZ00006045	2.64	down	flavonol glucosyltransferase
MZ00014291	4.52	down	phenylalanine ammonia-lyase
MZ00014292	13.01	down	phenylalanine ammonia-lyase
MZ00025088	2.88	down	phenylalanine ammonia-lyase
MZ00025089	3.48	down	phenylalanine ammonia-lyase
MZ00025513	3.52	down	cinnamic acid 4-hydroxylase
MZ00043784	5.58	down	cinnamic acid 4-hydroxylase

* (*P* < 0.05, 2 fold change as cutoff).

### 3.7. Regulatory Genes-Transcription Factors

Up-regulated transcription factors were investigated in the inoculated resistant and susceptible genotypes. Results indicated that R had fewer than S (Table S5). The gene with maximum fold-change in R was the DNA-binding protein RAV2 (MZ00017226, 4.3 fold). Many were down-regulated in S (not shown), but only one down-regulated factor was observed in R. Among the up-regulated TFs (totaling about 190), only four were expressed in both genotypes, including development regulation gene OsNAC4 (MZ00026127), DNA-binding protein RAV2-like (MZ00017226), ethylene responsive element binding factor (ERF) 3 (MZ00018574), and zinc finger transcription factor ZF1 (MZ00056566). 

Fewer TFs were shown to be significantly different between the R and S controls (Table S5). Most of the fold-changes in the expression of TFs were less than 4, and the maximum was 5.3. However, many transcriptional factors were shown to be significantly different between inoculated R and S. The maximum change was 25 fold. This indicates that TFs in R and S responded differently to challenge. Several TFs were shown to be significantly different in the comparisons of inoculated treatments and their controls, such as OSJNBb0020J19.6 (MZ00027110), unnamed protein product (MZ00024145), transcription initiation factor IIE (MZ00026843) in R, ethylene-responsive factor-like protein 1 (MZ00030445), and Sip1 protein (MZ00041367) in S. 

Of NPR1 gene members, only one (MZ00019046) was expressed in the inoculated R and S, but its expression was not significantly different between the inoculated samples and the controls. Of the WRKY genes, the WRKY9 (MZ00042052, MZ00042053, MZ00016272) and WRKY12 (MZ00021479) were expressed in both inoculated R and S, but expression was not significantly different between them. However, WRK (MZ00001709) and WRK12 (MZ00042508) were up-regulated significantly in the inoculated S. Many bZIP members were expressed in both inoculated R and S, such as MZ00043889, MZ00016963, MZ00028410, but none were up-regulated. A similar situation was observed with Myb genes; many members were expressed, but only Myb-like DNA-binding protein (MZ00044429, MZ00018761), and GAMYB-binding protein (MZ00024498) were up-regulated in inoculated S. Of the ethylene responsive factors, ERF3 (MZ00018574, MZ00026596) was up-regulated in inoculated R. ERF (MZ00016032), ethylene-responsive factor-like protein 1(MZ00019568, MZ00030445), and ERF3 (MZ00018574) were up-regulated in inoculated S.

Other transcription factors also were observed in inoculated R and S, such as zinc finger transcription factor ZF1 (MZ00056566), transcription factor MYC7E (MZ00044532), and AP2 domain factors.

### 3.8. Regulatory Genes-Signaling Pathways

From the survey of genes in signal biosynthesis pathways and down-stream response factors, a number of key ethylene pathway genes were expressed in the *A. flavus-*challenged samples. Some related genes were up-regulated, such as ACC oxidase (MZ00018436), ERF3 (MZ00018574, MZ00026596) in R; ACC synthase (MZ00018872), ERF3 (MZ00018574), ethylene-insensitive-3-like protein (MZ00042402, MZ00042403), ethylene-responsive factor-like protein 1 (MZ00019568, MZ00030445), ethylene-forming enzyme (MZ00027365), and ethylene-inducible CTR1-like protein kinase (MZ00025350) in S. Comparing inoculated R and S, the ethylene induced protein kinase PK12 (MZ00041589, MZ00001435) and ethylene receptor (MZ00025470) were up-regulated in R, but ethylene-forming enzyme (MZ00004140, MZ00027365) and ethylene-responsive factor-like protein 1 (MZ00030445) were down-regulated.

Besides ethylene, a number of key auxin pathway genes were also expressed in *A. flavus*’ challenged samples. Some auxin related genes were up-regulated in inoculated S, such as auxin response factor 1 (MZ00024113, MZ00024115), auxin response factor 2 (MZ00014879), auxin response factor (MZ00016434), auxin response transcription factor (ARF6) (MZ00018657), auxin-induced protein (MZ00055925), and auxin-regulated protein (MZ00017133). But in inoculated R, only the proliferating cell nuclear antigen (MZ00020357) was up-regulated by 2.3 fold. Compared with inoculated S, only auxin-regulated protein-like (MZ00042957) and auxin-induced protein (MZ00029389) were up-regulated by 25.0 fold and 8.3 fold respectively, and auxin response factor 2 (MZ00014879) and proliferating cell nuclear antigen (MZ00020357) were down-regulated by 2.0 fold and 4.8 fold respectively in R. 

In the present study, several genes in lipid metabolism were up regulated in the inoculated samples, such as lipase (MZ00037469), lipid transfer protein (MZ00041203, Z00041204), and membrane lipoprotein lipid attachment site-containing protein (MZ00001596) in R; lipoxygenase (MZ00015701, MZ00000521, MZ00041271), lipase-like (MZ00026059), lipid transfer protein (MZ00023565, MZ00041610, MZ00041611), and GDSL-motif lipase/hydrolase protein (MZ00005177) in S. Of the lipoxygenase isoforms, only MZ00041271 was up-regulated (4.0 fold) in the noninoculated R and S comparison, and only MZ00015701 was down-regulated (8.4 fold). Of the lipid transfer protein isoforms, the up-regulated genes were MZ00023565 (4.7 fold), MZ00019645 (3.9 fold), MZ00041613 (3.0 fold), MZ00041611 (3.4 fold) in this comparison; no down-regulated genes were observed. In the comparison between inoculated R and S, only up-regulated genes were detected. These include MZ00041612 (3.7 fold), MZ00041611 (4.7 fold), MZ00041613 (2.4 fold) and MZ00028450 (6.5 fold). The members of MZ00041611 and MZ00041613 displayed higher expression in both the inoculated R and the control. Lipid transfer proteins are also considered PR proteins. 

Of the kinases differentially expressed in the comparison between R and S controls, protein kinase Xa21 (MZ00001132) was up-regulated by 21.6 fold, LRR receptor-like kinase 2 (MZ00031205) by 2.7 fold, and receptor protein kinase (MZ00031498) by 2.6 fold. In the comparison between inoculated R and S, several were up-regulated such as protein kinase Xa21 (MZ00001132) by 27.0 fold, protein kinase A. FLAVUSC3 (MZ00015865) by 12.1 fold, and serine/threonine protein kinase (MZ00030536) by 15.1 fold. Several were also down-regulated, such as Avr9/Cf-9 rapidly elicited protein-like (MZ00041362) by 2.4 fold, MAP3K-like protein (MZ00018666), MAP kinase 4 (MZ00027390), casein kinase (MZ00013631) by 10.3 fold, serine/threonine protein kinase PKPA-like protein (MZ00056607) by 10.8 fold, and protein kinase (MZ00044579) by 11.6 fold. 

### 3.9. Validation of Microarray Data by Quantitative Real-Time RT-PCR

To confirm the reliability of the microarray results, twenty-four genes with the expression patterns of up-regulation, down-regulation, or no-change from microarray analysis were selected for validation using qRT-PCR (Table S6). Statistical significance and fold change based on relative quantification of C_T_ were analyzed for the selected genes in the comparison of inoculated R and S. Generally, *R*-value > 2.00 (*P* < 0.05) was described as up-regulation (++), *R*-value < 0.50 (*P* < 0.05) as down-regulation (−−), and 2.00 > *R*-value > 0.50 (*P* > 0.05) as no-change (+−). Results (Table S6) indicated that the expression patterns measured by qRT-PCR matchedthose measured by microarray, with regard to up-regulation, down-regulation and genes where no change occurred. The differences observed between the two methods were in expression level (fold change).

## 4. Discussion

KSA based *A. flavus*’ inoculation and incubation of kernels provides an efficient assessment method for aflatoxin accumulation. During this protocol, quiescent dry seeds will commonly germinate after the uptake of water. Different germination phases have been described, each with a unique metabolism status as well as gene expression pattern [33,34]. These phases include: (1) seed imbibitions; (2) reinitiation of metabolic processes; and (3) emergence of the radicle through the seed envelope [35]. KSA-processed kernels, which imbibe under 100% humidity, take longer to enter the third phase than do kernels steeped in water. It is also our observation, that the time required for radical emergence of incubated kernels varies with genotype. In some lines, radicals cannot be observed even past a 7-day incubation period. 

In the present study, R and S kernels were incubated via the KSA, 72 h for microarray analysis and 7 days for aflatoxin measurement. No radicle emerged by the 72 h time-point, however, variation in the amount of *A. flavus* colonization between R and S kernels was observed by this time-point. This variation facilitated the removal of kernels that differed from the resistant or susceptible phenotype (based on fungal growth on the kernel surface) used for the microarray experiment, therefore, avoiding the inclusion of false information in the microarray analysis. From the gene expression profiles of the two controls, metabolic processes in kernels had been reinitiated at 72 h. Therefore, the physiological status of the KSA kernels by definition would be phase 2. Microarray results indicate that kernels could sense and respond to a challenge from *A. flavus* at this stage, as a complex defense system was initiated in response to *A. flavus* infection in both R and S lines. Also, multiple defense genes were shown to be involved in this system. 

In general, the comparison between resistant and susceptible controls demonstrated that the total expressed genes and their biological processes have similar expression patterns (Figure 1); transcriptional profiles of imbibed kernels at the early phase of germination are also similar to profiles of inbred Tex6 kernels during late development in the field [26]. This result suggests that imbibed kernels at the early germinating stage are restored to the physiological status existing prior to kernel dormancy. Further studies, however, comparing gene expression during imbibition with expression in late development, within the same genotype, would be required to confirm this suggestion. Imbibed kernels (early germination) might then provide a more suitable subject for gene expression analysis than late developing kernels from the field. To the authors’ knowledge, the present study represents the first time a gene expression profile has been obtained using imbibed kernels to investigate the maize-*A. flavus* interaction. Since aflatoxin-resistance in pre- and in post- harvest kernels correlates well [12,13], using imbibed kernels may also facilitate further understanding of the ability of mature pre-harvest seed, where aflatoxin buildup occurs in the field, to respond and defend against *A. flavus* infection and aflatoxin production. 

All plants have a basal defense, the general immune response to pathogens and other mechanisms to counter microbial infections [19,22,36]. Earlier proteomic investigations demonstrated that possession of a strong constitutive resistance is a primary factor differentiating resistant from susceptible kernels [23]. By comparing the gene expression profile in R and S control kernels, numerous defense genes were clearly detected, and these genes could be part of the normal kernel development process under germinating conditions and a part of the constitutive resistance against potential pathogens and environmental stress at this stage of development. However, different genotypes could have different defense genes or different expression levels, even genotypes with close genetic backgrounds, such as Eyl25(R) and Eyl31(S), which are 87.5% genetically similar (Table 1). The comparison between R and S controls shows that more PR genes were expressed in R than in S. Several members of the chitinase family were up-regulated significantly in R, however, none were up-regulated in S. Chitinase may play an important constitutive defense role in R; one member, PR-4 (MZ00043659), was expressed by 36.3 fold higher in R than in S.

In response to challenge by *A. flavus*, defense genes were induced in both aflatoxin*-*resistant and susceptible genotypes, especially PR genes. Most PR gene families were observed in both genotypes after induction, but the members and their expression levels varied between the two maize lines. Some induced PR genes in inoculated samples were also highly expressed in controls, such as PR4 (MZ00043659), beta-1,3-glucanase (MZ00030174), zeamatin (MZ00017927), and nonspecific lipid-transfer protein (MZ00041611). An interesting result was that no PR gene was down-regulated in the inoculated R. The gene expression profiles of both genotypes revealed that S kernels were even more sensitive to challenge by *A. flavus* than R kernels. A proteomic investigation of aflatoxin-resistant and -susceptible maize rachis tissue showed the same variation in sensitivity to challenge by *A. flavus* observed in the present study [37]. The response of S and R genotypes to infection presumes the presence of a recognition and regulation system in kernels. Based on this presumption, kernels under attack would determine the defense components needed to ward off a pathogen. The lack of adequate preformed components could therefore, lead to the expression of numerous genes for defense purposes. On the other hand, the resistant line, with ‘adequate’ constitutive resources would be less sensitive in its response to pathogen attack, synthesizing components to a lesser degree than the susceptible line. This study and future investigations may assist us in understanding an “*A. flavus* recognition and defense-response system”. This phenomenon may provide a new strategy for screening lines for resistance at the molecular level. 

Since numerous constitutive and induced genes comprise maize kernel resistance to *A. flavus* infection, devising a sound defense strategy may require an understanding of the regulation network involved in the kernel defense response. The discovery of transcription factors (TFs) expressed in response to challenge could help in understanding the regulation of defense genes and the response of TFs to signal transduction in kernels. Besides constitutive TFs in the noninoculated R and S, many TFs were induced in response to infection, and differences in induced TFs between R and S kernels were demonstrated (Table S5). In plant disease resistance, transcription cofactor NPR1 controls the expression of antimicrobial PR genes by interacting with other transcription factors, such as WRKY, ERF, bZIP, Whirly and Myb factors [22,36,38,39]. WRKY factors appear to play a major role in transcriptional reprogramming during a variety of immune responses [40]. One NPR1, several WRKY and many bZIP members were expressed in the inoculated R and S, however, no up-regulated ones were observed in the comparison of controls. Of the observed Myb and ERF members, three Myb and four ERF members were up regulated in the inoculated S, no Myb members but two ERF members were up regulated in the inoculated R. So, ERF members could be involved in the response of R and S kernels to *A. flavus*, especially ERF3 (MZ00018574), expressed in both inoculated genotypes. ERFs are known to comprise one of the largest families of transcription factors in plants, and play a virtual role in response to biotic and abiotic stress. In response to pathogen infection, ERF proteins activate the expression of PR genes by binding to the GCC box (AGCCGCC) in the promoter of PR genes, which positively regulates resistance to pathogen attack [41,42,43].

Several plant signaling components have been shown to be involved in the induction of plant defense, such as salicylic acid, jasmonic acid, ethylene and reactive oxygen species [22,36,44]. Research also suggests that a lipid-based molecule could be the mobile signal in plant defense systems [36]. Interestingly, previous evidence implicates a lipid metabolite as playing a signal role in host resistance against *A. flavus* infection [45,46,47]. In fact, different isoforms of lipoxygenase, could lead to different host responses to *A. flavus* infection. The present microarray investigation of signaling components indicate that ethylene, auxin, and lipid pathways are involved in the response to challenge by *A. flavus*. The relationship between the related pathways, however, still must be uncovered. 

Receptors and kinases are important signal transduction components in plant defense systems [22]. Of the detected receptors and kinases, Avr9/Cf-9 rapidly elicited protein, mlo2 protein and receptor-like kinase Xa21-binding protein 3 are expressed in both R and S controls. Avr9/Cf-9 rapidly elicited protein and mlo2 protein have been shown to be involved in resistance to fungal pathogens [48,49]. Xa21 serves as a pathogen recognition receptor in rice to innate immune systems in resistance to bacterial blight disease caused by *Xanthomonas oryzae* pv. *Oryzae* [50]. Interestingly in the comparison between R and S, protein kinase Avr9/Cf-9 rapidly elicited protein-like structure (MZ00041362) was up-regulated in the inoculated S, and Xa21 (MZ00001132) was significantly higher in both the control and the inoculated R than in S samples. However, further work is needed to determine the exact involvement of Xa21 (MZ00001132) or the Avr9/Cf-9 rapidly elicited protein-like structure (MZ00041362) in the interaction with *A. flavus*, and if higher expression of Xa21 in R accounts for the difference in *A. flavus* resistance between R and S. 

## 5. Conclusions

The present gene expression study of closely-related maize lines, aflatoxin-resistant Eyl25 and susceptible Eyl31, which vary in aflatoxin accumulation, displays a network of genes expressed, with and without challenge by *A. flavus*. This includes the identification of regulatory genes and their differential expression between resistant and susceptible phenotypes. By analyzing the gene expression profile, the relationship between genes and their products can be determined, on a quantitative and qualitative level. This research can aid in understanding kernel resistant mechanisms at the transcription level, and assist in the discovery of target genes for enhancing resistance in maize. The use of imbibed mature kernels as microarray subjects in the present study, offers researchers a potentially quicker and easier way of obtaining kernel materials for profiling genetic differences while controlling environmental factors to a greater degree than previously achieved using traditional methods.

## Supplementary Files

Table S1 Defense related genes expressed in controls

Table S2 Differential expression of defense-related genes in the comparison of Eyl25 and Eyl31 Controls

Table S3 Defense genes expressed in A. flavus infected Eyl25 and Eyl31

Table S4 Comparison of defense gene expression between inoculated Eyl25 and Eyl31

Table S5 Transcription factors expressed in comparisons of all treatments

Table S6 Validation of results by RT-PCR
